# The Assessment of the Risk of Malnutrition (Undernutrition) in Stroke Patients

**DOI:** 10.3390/nu15030683

**Published:** 2023-01-29

**Authors:** Olivia Di Vincenzo, Maria Luisa Eliana Luisi, Paola Alicante, Giada Ballarin, Barbara Biffi, Chiara Francesca Gheri, Luca Scalfi

**Affiliations:** 1 Human Nutrition and Dietetics, Department of Public Health, Federico II University, 80131 Naples, Italy; 2 IRCCS Fondazione Don Carlo Gnocchi ONLUS Firenze, 50143 Florence, Italy; 3 Department of Movement Sciences and Wellbeing, Parthenope University, 80133 Naples, Italy; 4 Casa di Cura Santa Maria del Pozzo, Somma Vesuviana, 80049 Naples, Italy

**Keywords:** malnutrition, nutritional screening, stroke, rehabilitation, MUST, NRS-2002, MNA, GNRI, PNI, CONUT

## Abstract

Malnutrition is common in stroke patients, as it is associated with neurological and cognitive impairment as well as clinical outcomes. Nutritional screening is a process with which to categorize the risk of malnutrition (i.e., nutritional risk) based on validated tools/procedures, which need to be rapid, simple, cost-effective, and reliable in the clinical setting. This review focuses on the tools/procedures used in stroke patients to assess nutritional risk, with a particular focus on their relationships with patients’ clinical characteristics and outcomes. Different screening tools/procedures have been used in stroke patients, which have shown varying prevalence in terms of nutritional risk (higher in rehabilitation units) and significant relationships with clinical outcomes in the short- and long term, such as infection, disability, and mortality. Indeed, there have been few attempts to compare the usefulness and reliability of the different tools/procedures. More evidence is needed to identify appropriate approaches to assessing nutritional risk among stroke patients in the acute and sub-acute phase of disease or during rehabilitation; to evaluate the impact of nutritional treatment on the risk of malnutrition during hospital stay or rehabilitation unit; and to include nutritional screening in well-defined nutritional care protocols.

## 1. Introduction

As specified by the European Society for Clinical Nutrition and Metabolism (ESPEN) [1], malnutrition (undernutrition) can be defined as “a state resulting from lack of intake or uptake of nutrition that leads to altered body composition (decreased fat-free mass) and body cell mass with diminished physical and mental function and impaired clinical outcome from disease”. As a consequence, a poor nutritional status negatively affects diagnosis, prognosis, and the clinical course of various acute or chronic diseases [2].

Stroke represents a leading cause of death worldwide [3], wherein acute ischemic stroke (AIS) is more prevalent than hemorrhagic (AHS) stroke, and it is also a primary cause of temporary or chronic disability, precipitating important consequences in social and economic terms [4,5]. Malnutrition is common in stroke patients (in most cases, elderly individuals), who often also suffer from comorbidities such as diabetes mellitus, heart disease, neurological and cognitive deficits, depression, infections, etc. [4,5,6,7]. However, the prevalence of malnutrition after stroke varies widely, which is potentially due to patients’ characteristics (for example, age or type of stroke) as well as the diagnostic procedures used [4,5]. Indeed, it is widely accepted that malnutrition affecting stroke patients is negatively associated with several clinical outcomes in both the short- and the long-term period, wherein the foremost concerns are disability and mortality [4,5,6,7].

In the clinical setting, nutritional screening and the assessment of nutritional status are both fundamental components of the nutrition care of patients [1,2,6,8,9,10]. Nutritional screening is the process used to identify patients at nutritional risk (i.e., risk of malnutrition) or who may be at risk, while nutritional assessment is a more detailed approach used to attain and interpret data in order to identify nutrition-related problems and their causes; this approach yields significant results in terms of severity grading, diagnosis, prognosis, and treatment. Unfortunately, despite its relevance to both prevention and treatment, implementing best practices in nutritional care is often not adequately considered in the multidisciplinary approach to the diagnosis and management of patients affected by acute or chronic diseases [1,2,8].

A realistic first step would be to identify a reliable and proper (i.e., formal) approach for assessing nutritional risk, which should be validated, rapid, simple, cost-effective, reliable, and helpful in the clinical setting. There are many tools/procedures for nutritional screening in use across the globe, and several have been applied to stroke patients [5]. Some of them initially account for information on body mass index (BMI), changes in body weight, or reduced food intake, which are expected to be related to nutritional status [2,8]. Basic laboratory tests, such as albumin levels, are included in other tools, possibly being associated with inflammatory status and clinical outcomes [2].

Overall, evaluating the evidence on nutritional screening may help identify the best practices with respect to implementing a nutritional care program. Thus, we carried out a review based on a consistent analysis of the literature, focusing on the tools/procedures applied to stroke patients as a formal approach for assessing the risk of malnutrition, with a particular interest in their relationships with patients’ clinical characteristics and outcomes. Different settings and types of strokes as well as various clinical outcomes have also been taken into account.

## 2. Methods: Literature Search Strategy

This is a literature review regarding the nutritional screening tools used in stroke patients. A bibliographic search was carried out in PubMed and checked by examining SCOPUS, Embase, and Web of Science. According to an initial search using the terms “nutritional status”, “nutritional risk”, and “risk of malnutrition”, the following tools were selected: the Malnutrition Universal Screening Tool (MUST), Nutritional Risk Screening 2002 (NRS-2002), the Mini Nutritional Assessment short form (MNA-SF), the Geriatric Nutritional Risk Index (GNRI), the Prognostic Nutritional Index (PNI), and the Controlling Nutritional Status score (CONUT). Other tools/procedures (Full MNA, Subjective Global Assessment (SGA), American Society for Parenteral and Enteral Nutrition-ASPEN and ESPEN criteria, and Global Leadership Initiative on Malnutrition (GLIM) criteria) were not considered because they were not designed for screening but for a definite assessment/diagnosis of malnutrition [2]. Thus, we applied a search strategy using the following terms as strings in full texts: “Nutritional screening AND stroke”, “Malnutrition AND stroke”, “(Malnutrition Universal Screening Tool or MUST) AND stroke”, “(Nutritional Risk Screening 2002 or NRS-2002) AND stroke”, “(Mini Nutritional Assessment or MNA) AND stroke”, “(Geriatric Nutritional Risk Index or GNRI) AND stroke”, “(Prognostic Nutritional Index or PNI) AND stroke”, and “(Controlling Nutritional Status or CONUT) AND stroke”. 

The search was limited to reports in English without any limitations regarding the year of publication. The eligibility criteria were as follows: (1) original studies on patients with AIS and/or AHS (including subarachnoid hemorrhage (SAH)); (2) assessment of the risk of malnutrition using one of the aforementioned tools; and (3) results published as full papers in peer-reviewed journals. We also searched the texts and reference lists of retrieved studies to identify additional publications.

## 3. Nutritional Screening Tools

The main information regarding the included studies assessing nutritional risk among stroke patients is reported in Table 1 and Table 2. Table 1 indicates the setting, the type of stroke, and the tool(s) used in each paper, while Table 2 summarizes the principal clinical outcomes related to nutritional risk, which are mortality, disability/independence in activity of daily living-ADL, infections, length of hospital stay (LOS), dysphagia, and cognitive impairment. We do not consider sparse evidence available for other tools such as the HALP (hemoglobin, albumin, lymphocyte, and platelet) score [11,12] or short nutritional assessment questionnaire (SNAQ) [13].

The paragraphs that follow contain information on each tool in relation to its main characteristics, its use in stroke patients, the evaluation of nutritional risk, and the relationships with clinical outcomes.

### 3.1. Malnutrition Universal Screening Test (MUST)

MUST was designed in 2003 by The British Association for Parenteral and Enteral Nutrition (BAPEN) to help identify adults at risk of undernutrition [2]. According to this procedure, patients are classified into risk levels based on BMI (>20 kg/m² = 0; 18.5–20 = 1; <18.5 = 2), unintentional weight loss in the previous 3–6 months (<5% = 0; 5–10% = 1; >10% = 2), and the presence of an acute disease plus a reduction in food intake for >5 days (absence = 0; presence = 2). Overall malnutrition risk is calculated by adding the scores obtained for each of the three components; a score of 0 indicates low malnutrition risk, a score of 1 indicates medium risk, and a score of ≥2 corresponds to high risk. 

Table 1 shows that the studies using MUST were carried out in the United Kingdom, China, and Japan in stroke patients (mostly with AIS) admitted to the hospital or to a rehabilitation unit [21,24,50,65,70]. The risk of malnutrition ranged between 22–37% in the four studies in hospitalized patients and rose to 51% in the study on rehabilitation. 

In a well-structured study [24], nutritional risk at admission was related to several parameters, including a worse level of consciousness, infection rate, disability at discharge, palliative care, discharge to a new care home, and mortality. The other two papers showed that, in stroke patients, MUST predicts mortality during hospitalization, LOS, discharge to an acute care hospital, and hospitalization costs [21,50,65]. MUST also emerged as an independent predictor of mortality and poor functional outcome at 3 and 12 months [70].

### 3.2. Nutritional Risk Screening 2002 (NRS-2002)

NRS-2002 is an ESPEN-recommended screening tool for hospitalized patients [2]. It has an initial screening phase with four questions: BMI < 20.5 kg/m², weight loss in the last 3 months, reduced food intake in the last week, and severe illness. If the answer is “yes” to any of these questions, the screening phase is performed: (1) based on body weight loss (at least >5% in three months), BMI, and reduced food intake, a score of 0 to 3 is assigned; (2) according to disease severity (current clinical conditions and the presence of diseases affecting nutritional status), a score of 0–3 points is also assigned; finally, 1 point is added for patients aged ≥ 70 years. A score <3 indicates no risk of malnutrition, while a score ≥3 indicates a high nutritional risk, indicating the need for nutritional support. 

As shown in Table 1, the studies using NRS-2002 on AIS patients (from 2020 to 2022) were carried out in China on hospitalized patients [17,19,39,70]. The proportion of patients at risk of malnutrition was around 35–50%.

At baseline, the patients with high nutritional risk had lower independence in to ADL and hemoglobin levels, higher D-dimer concentrations, and greater severity of disease compared to the others [7]. In addition, an NRS-2002 score ≥3 has been associated with the development of stroke-associated infections during hospitalization, the need for nutritional support and LOS (Table 2), and ADL at discharge. In the long-term period, a high nutritional risk was found to be a predictive factor of mortality and poor functional outcomes at 3 [17] and 12 months [70] post-discharge; this latter association was also confirmed by Liu et al. [39] among stroke patients with dysphagia.

### 3.3. Mini Nutritional Assessment Short Form (MNA-SF)

The MNA-SF is the short form of the MNA, which is widely used for older people. While the MNA-SF is used as a nutritional screening tool, the full form is used for nutritional assessment. This short form includes six elements (also included as the first section in the full MNA): current BMI, recent loss of weight, presence of acute disease or stress, bedridden status, the presence of dementia/depression, and loss of appetite/difficulty eating. The interpretation criteria are as follows: a good nutritional status is assigned for a score ≥12 and a risk of malnutrition is assigned for scores of 8–11, while it is possible to proceed to a direct diagnosis of malnutrition if the score is ≤7 [2].

The studies that applied the MNA-SF to stroke patients were all carried out in Far Eastern Countries, apart from one from Italy [18,34,35,49,50,51,52,62,63], as shown in Table 1. In most studies, patients (suffering from AIS or AHS) were recruited by rehabilitation facilities. The studies commonly used the cut-off values proposed in the original paper, but a new reference range of 5–7 was also derived and used to define nutritional risk [50].

Upon admission to the rehabilitation ward, a substantial proportion (30–40%) of patients were at risk of malnutrition [50,63]. In addition, the MNA-SF score was lower among patients identified as malnourished using GLIM [62] or among those who were sarcopenic [63].

Upon admission, the MNA-SF score is significantly related to cognitive impairment (Table 2) and independence in ADL [35,49,51,52], but not quality of life [35]. The MNA-SF showed better improvement when energy intake upon admission was higher [52], and when a post-acute care program was compared to a traditional inpatient rehabilitation approach [18]. As for clinical outcomes, in a rehabilitation setting, the MNA-SF has been associated with functional independence at discharge but not with the discharge destination [50,51]. There was also a significant relationship between nutritional risk upon admission and post-stroke cognitive impairment [34].

### 3.4. Geriatric Nutritional Risk Index (GNRI)

The GNRI score, commonly used for elderly individuals and critically ill patients [2], is calculated as follows: GNRI = 14.89 × albumin (g/dL)] + 41.7 × (weight/ideal body weight). In the original paper, four levels of nutritional risk (categories) were proposed: a major risk for GNRI <82, a moderate risk for GNRI 82 to <92, and a low risk for GNRI 92 to ≤98, with normal values for GNRI >98.

The studies that applied GNRI to stroke patients have been carried out (since 2016) in Far Eastern Asian countries [7,14,15,16,27,29,30,31,32,34,37,42,43,45,46,47,48,50,53,54,55,57,58,63,64,66,68,70,71], as shown in Table 1. A significant risk of malnutrition has usually been defined by a GNRI score <92, with some exceptions (for example, a score <98 [7] or <100 [27]).

GNRI was determined upon admission to hospital or a rehabilitation unit (Table 1) in patients aged 65 and over [70], but occasionally in the younger ones as well [42]. Two papers reported data on GNRI changes after rehabilitation [57,63]. Results are available for stroke patients with AIS and/or AHS (including also SAH); indeed, much more information is available on AIS patients.

Most studies reported data on the distribution of nutritional risk. Mean values of GNRI score widely varied from a minimum of 87 at admission to a rehabilitation ward [47] to a maximum of 106 in hospitalized patients [34]. A very high prevalence of moderate–severe risk (score <92) was observed in a multicenter study performed in rehabilitation wards [48], with lower values (usually in the range 10–30%) being reported in hospitalized patients. As expected, a higher proportion of patients at risk was found when higher cut-offs (see above) were chosen [7,27,42].

In elderly stroke patients, GNRI (in parallel with BMI and albumin) decreased during hospitalization, and these changes were related to age, AIS, severity of disease, and independence in ADL [57]. Furthermore, a decline in GNRI (i.e., an increase in nutritional risk) was also observed over rehabilitation periods [57,63]. The improvement in GNRI after rehabilitation was associated with greater independence in ADL [29,47], but these findings were not confirmed by a more recent study [30].

According to the small pool of published information, an association of GNRI with pneumonia incidence was observed in hospitalized stroke patients with dysphagia [48] or diabetes mellitus [64], but was not confirmed in another study [14]. GNRI was also a significant predictor of early neurological deterioration [15], transferal from rehabilitation to acute care hospital units [50], post-discharge destination (home or transfer) [57], or the ability to achieve adequate oral intake [48].

As for long-term outcomes, an association between poor functional outcome and low GNRI (as score or categories) has been reported by several studies (Table 2), with specific information available for AIS but not for AHS patients. In one paper [70], malnutrition risk was associated with functional recovery and mortality after a 3-month follow-up but not after 12 months, while in another study [68] GNRI was still predictive of mortality after 6 years. A recent meta-analysis [73] based on five studies showed that low GNRI is an independent predictor of poor functional outcome even if only AIS patients were taken into consideration. A low GNRI was independently associated with infectious diseases during a one-year follow-up [42] and with the development of post-stroke cognitive impairment at 3 months after ischemic stroke [37].

Finally, a significant association between GNRI and mortality was observed in several papers (Table 2) over different follow-up periods ranging from 3 to 56 months after stroke. Once more, according to the aforementioned meta-analysis [73], all-cause mortality was associated with low GNRI (five studies). Furthermore, among patients discharged from a rehabilitation ward and followed up for more than 1 year, GNRI was an independent determinant not only of all-cause mortality but also of cardiovascular events [68].

### 3.5. Prognostic Nutritional Index (PNI)

The PNI score is calculated according to the following formula: PNI = 10 × serum albumin (g/dL) + 0.005 × total lymphocyte count (n/mm³). According to the original paper, a score >38 reflects a normal nutritional status, with scores of 35–38 and <35 indicating moderate and severe risk of malnutrition, respectively [2]; in this case, there is no “mild/low” category. This classification has been used by most papers applying the PNI to stroke patients. Indeed, one study defined two categories of the PNI, namely, high PNI (>44.15) and low PNI (44.15), as indicated by a ROC analysis considering functional outcomes [67], while in other papers the cut-off for low PNI was <39.7 [38] or tertiles were used to categorize patients [25].

As shown in Table 1, the studies were carried out in AIS patients (SAH in one) in Far Eastern Asian countries, except one in Italy [15,25,28,38,41,44,56,59,65,66,67,68,71]. All the studies but one involved stroke patients at admission to hospital (Table 1).

Using the PNI, there was a prevalence of moderate/severe nutritional risk ranging from 2% [71] to 31% [68]. The PNI at baseline has been associated with age, male sex, smoking, clinical characteristics (hypertension, atrial fibrillation, previous stroke, and anemia) and nutrition-related factors such as BMI, total cholesterol, albumin, lymphocyte count, and hemoglobin concentration [67]. Among elderly AIS patients, the PNI was related to short-term outcomes such as early neurological deterioration [15]. As for long-term (≥3 months) outcomes, a low PNI was found to be an independent predictor of functional outcome and lower independence in ADL at three months (Table 2) as well as fatigue at six months [28]. Furthermore, findings showed that the PNI was an independent predictor of mortality among acute patients (Table 2) but not of mortality or transfer to acute care among patients undergoing rehabilitation [59]. Lastly, the PNI was related to a lower risk of long-term recurrent AIS and major cardiovascular events [25].

### 3.6. Controlling Nutritional Status (CONUT)

The CONUT scoring system, proposed as a screening tool for hospitalized patients [2], is calculated from serum albumin concentration, total peripheral lymphocyte count, and serum total cholesterol concentration. A score is first derived for each of the parameters in comparison to the corresponding normal range (i.e., evaluating the extent of decrease). Based on the total score, the patients are classified as having normal (score 0–1), light (2–4), moderate (5–8), or severe (9–12) risk of malnutrition.

All the studies applying the CONUT tool to stroke patients were carried out (since 2018) in Far Eastern Countries, with the exception of one from Spain [14,15,17,20,22,23,25,26,30,33,34,36,40,41,44,45,46,56,60,61,66,67,68,69,70,71,72], as reported in Table 1. Stroke patients were classified according to the original four, but also in three (normal, light, and moderate/severe risk) or in two (absence or presence of risk) categories; a CONUT score ≥5 was commonly defined as the cut-off for a significant risk of malnutrition, but in two papers the cut-off was ≥2 [72] or ≥4 [23], respectively. 

The studies involved patients at admission to hospital while two were carried out in inpatient rehabilitation facilities (Table 1). CONUT was evaluated at admission with only one study reporting data at discharge [22]. By far, most studies evaluated AIS patients; two also included AHS patients and one only included patients with SAH (Table 1). The prevalence of moderate/severe risk of malnutrition (score ≥5) among hospitalized patients ranged from 4.6 [60] to 18.2% [44], with a much higher figure when a cut-off of 2 was used (52.4%) [72], and with a negligible proportion (0.5 to 8%) of patients with a score 9–12. In rehabilitation wards, the proportions of stroke patients at moderate/severe risk of malnutrition were 16 and 40% [30,36].

Upon admission to a hospital, CONUT categories/score have sometimes been related to various parameters such as tooth loss, motor function at admission, dysphagia, consciousness disorders, hemoglobin, and anemia [14,17,33,60,68,70]. 

Outcomes related to hospitalization and discharge have been considered by a few papers, showing that CONUT score/categories were associated with in-hospital mortality, LOS, infection, nutritional support, length of rehabilitation, and independence in ADL at discharge [17,26,33,70,72]. One study [26] evaluated AIS and an age-matched control group represented by hospitalized patients admitted to a hospital for disorders not related to acute stroke; it reported higher CONUT scores, higher in-hospital mortality, and longer LOS in AIS patients. In a convalescent ward, CONUT score was a predictor of the length of stay but not of changes in functional independence [30]. Among non-clinical outcomes, CONUT score was associated with hospitalization costs [33]. 

CONUT score has been related to various long-term (≥3 months) outcomes, with most data based on a three-month follow-up. A low CONUT score was directly associated with functional outcome and lower independence in ADL (Table 2), and it was also identified as an independent predictor of infection (pneumonia and urinary tract infection) during hospitalization or at 3/6 months after stroke (Table 2). Furthermore, CONUT score was associated with long-term recurrent stroke and major cardiovascular events [25] and was an independent prognostic factor of mortality after a follow-up of 3 to 12 months [70]. Regarding clinical characteristics, Naito et al. [45] evaluated CONUT score in different ischemic stroke subtypes (small-vessel occlusion, large-artery atherosclerosis, cardioembolic stroke, and other etiologies), showing that patients with CONUT scores ≥5 who had had cardioembolic stroke and strokes of other etiologies had a significantly higher frequency of poor outcomes than those with CONUT scores <5.

### 3.7. Comparison between Tools

Several studies assessed the risk of malnutrition using different screening tools in the same paper, for example, both GNRI and CONUT (Table 1). Overall, the prevalence of nutritional risk widely varied depending on the tool used; in recent papers, it was as follows: GNRI 34%, PNI 7%, and CONUT 10% [66]; GNRI 32%, PNI 31%, and CONUT 8% [70]; MUST 37%, NRS-2022 45%, GNRI 32%, and CONUT 10% [68]; and GNRI 5%, CONUT 6%, and PNI 2% [71].

Nearly all tools have been applied to hospitalized patients. MNA-SF is the only one that has been used more frequently (seven out of nine papers) in rehabilitation than hospitalized patients; one third of studies using GNRI have also been carried in rehabilitation units.

As for clinical outcomes, risk of malnutrition assessed by either CONUT, GNRI, or PNI has been associated with significantly increased risk of mortality or disability (Table 2). In addition, CONUT, GNRI, and PNI were predictors of early neurological deterioration with a similar discriminative ability for all the tools [15]. Both CONUT and PNI proved to be promising screening indicators with respect to identifying AIS patients at higher risk of long-term recurrent AIS and major cardiovascular events [25]; MNA-SF and GNRI showed fair concurrent validity with respect to predicting discharge destination [50]. On the other hand, one study found no relationship between CONUT or GNRI at admission and functional outcome at discharge [30]. CONUT score appeared to be more useful than GNRI or PNI for predicting a poor functional outcome at discharge [14] or at 3 months [41,44,45], while in another study, CONUT might have more efficiently predicted 3-month neurological outcomes after SAH than PNI [56]. Other findings showed that MNA-SF (but not GNRI or CONUT) might be associated with quadriceps muscle thickness change in non-paralytic limbs [34]. It has also been reported that NRS-2002 better predicts poor functional outcomes at 3 months than CONUT [17]. Finally, in a recent study in a large, multicenter cohort of AIS patients, CONUT, GNRI, MUST, and NRS-2002 were all associated with poor outcome at 3 months whilst only NRS-2002 and MUST were significantly associated with poor outcomes at 12 months post-discharge [70].

## 4. Discussion

In this literature review, we have evaluated studies regarding the assessment of nutritional risk in stroke patients (upon admission to hospital, during hospital stay, or during rehabilitation) in order to provide an overview of the tools/procedures to use as first steps in nutritional care planning. An in-depth analysis of each of these tools/procedures may be performed in further studies.

Stroke is not only a leading cause of death worldwide [3] but also causes disability (in both the short and long term), with AIS more common than AHS. The prevalence of malnutrition after stroke has been found to vary widely, possibly because, at least in part, of the diagnostic procedures used [4,5].

Alterations of nutritional status have been described in stroke patients in terms of body composition (i.e., fat-free mass or calf circumference), energy expenditure, and laboratory tests (for instance, vitamin status and plasma proteins such as transferrin and transthyretin) [4,5,74,75]. On the other hand, the best approach to apply in hospital or rehabilitation units for nutritional screening and the diagnosis of malnutrition remains to be defined, and these are both basic components of a nutrition care plan [1,2,6,8,9,10]. In particular, only in a few cases has a “formal” diagnosis of malnutrition been produced based on well-defined and shared criteria [5]; this is also true for sarcopenia [76].

In the present review, the assessment of nutritional risk by means of validated, rapid, simple, cost-effective, and reliable tools is considered as a first step in implementing an effective nutritional care program. Various tools/procedures for nutritional screening have been applied to stroke patients [5]. Some of them initially account for information on BMI, changes in body weight, or reduced food intake, which are expected to be related to nutritional status [2,8]. Basic laboratory tests, such as albumin levels, are included in other tools, possibly being associated with inflammatory status and clinical outcomes [2].

The next paragraphs summarize the main points that emerge from our data analysis.

Overall, choosing a screening procedure is a matter of balance between the healthcare setting, resources, type of patient, operational problems, the characteristics of the tool, etc. Some tools/procedures for assessing nutritional risk were proposed by expert panels [1,2,6,8,9,10] based on the relationships between malnutrition and weight loss, low BMI, decreased food intake, etc. Others include basic laboratory test data, of which the foremost component is albumin concentration, and were developed by examining the nutrition-related predictors of clinical outcomes [2]. Various tools/procedures have been applied for assessing the risk of malnutrition among stroke patients, with a sudden increase in the number of published papers over the most recent years (Table 1). Nutritional risk has been reported in terms of prevalence in the study groups as well as being related to patients’ clinical characteristics at baseline and outcomes in the short/long term. There have been few studies using MUST or NRS-2002, although these two tools are commonly proposed, and recommended by ESPEN, for hospitalized patients or the general population [2]. Data on MNA-SF, which is a specific approach for assessing the elderly [2], have also been reported in a limited number of papers (Table 1). On the contrary, GNRI and CONUT have become very popular and were frequently used in 2022 [2]. 

All tools have been applied to hospitalized patients, whereas GNRI and MNA-SF have been the two most frequently used in rehabilitation facilities (Table 1). It should be noted that some of them had been designed for and usually applied to hospitalized patients. On the contrary, MNA-SF seems to explore middle- or long-term changes in nutritional risk factors (i.e., weight loss, food intake etc.). 

Much more information is available regarding AIS or pooled AIS/AHS patients, with a relatively low number of studies involving only AHS or SAH patients or those submitted to intravenous thrombolysis [66,67]. So far, there has been no systematic analysis of the differences between sexes or different age groups. In most cases, the data were obtained at admission to hospital, with less evidence acquired during rehabilitation (Table 1). Of note, for all the tools (except MUST), most studies have been performed in Far Eastern Countries.

Regarding the screening tests that have continuous results (measured on a scale), cut-off values were used to subdivide the patients into different categories. Usually, the cut-offs proposed by the original papers were used. In others, new cut-offs have been derived by considering the relationships between scores and malnutrition or clinical outcomes. Statistical analysis has been performed by considering the scores as continuous variables (for example, with respect to GNRI or CONUT) or categories (i.e., light, moderate, or severe risk).

Overall, nutritional risk varies remarkably between studies and also between studies using the same screening tool. There are no definite data regarding the difference in nutritional risk between AIS and AHS, whereas a greater risk might be attributed to patients admitted to rehabilitation units [30,36,59,63] Indirectly, this suggests that nutritional risk may increase during hospitalization due to low food intake, inflammation, comorbidities, etc.

The relationships of nutritional risk with variables/outcomes of interest at baseline, during stay, or at discharge have been examined in different ways. As highlighted in Table 2, in the multidisciplinary approach to the treatment of stroke patients, the outcomes of interest are likely to differ depending on the setting. Among hospitalized patients, greater attention is expected to be focused on infections, LOS, dysphagia, mortality, and functional recovery, as well as the discharge destination. In the medium and long term (i.e., rehabilitation), the association of nutritional risk with cognitive impairment, mortality, and disability becomes more relevant. In stroke patients, the available evidence suggests that a greater nutritional risk is associated with infection, LOS, dysphagia, cognitive impairment, tooth loss, motor function at admission, discharge destination, etc. It is also worth mentioning that a greater nutritional risk has been observed in sarcopenic stroke patients [16,63], suggesting that both these conditions should be assessed at the same time in order to better describe patients’ nutrition-related problems.

The most convincing results have been gathered for long-term outcomes, as summarized in Table 2. Nutritional risk has been found to be a significant predictor of mortality and disability/independence in ADL, and these findings have also been confirmed via multivariate analysis. Most results have been produced using CONUT or GNRI, and often in retrospective studies.

Some papers have reported results for two or more screening tools used in the same group of patients (Table 1), but the agreement between methods has not been evaluated in detail. Available data suggest that the prevalence of nutritional risk is greater using GNRI (and possibly MNA-SF) than using CONUT and PNI, possibly depending upon the characteristics of each tool.

In practical terms, some comments may be made regarding specific screening tools/procedures. Some of them, such as MUST or NRS-2002, cannot easily be utilized to assess stroke patients (especially in the acute phase) because of the difficulties in collecting information on anthropometric measures, weight loss, food intake, and body composition. For the same reasons, MNA-SF might have been more frequently preferred in rehabilitation facilities. GNRI has been frequently applied to patients during rehabilitation, while it is likely that CONUT and PNI have become popular because of the ease of retrieving laboratory test data appropriate for retrospective analysis. Finally, it is interesting to note that according to NRS-2002, all stroke patients aged ≥70 should be considered at risk of malnutrition.

It is commonly accepted that a more comprehensive assessment of nutritional status has to be implemented in the assessment of patients at nutritional risk by measuring body composition, disease-specific nutritional markers, inflammation markers, muscle strength, etc., or using other formal approaches such as full MNA or GLIM criteria [2]. Overall, this has not been the case of the papers retrieved for this review. In addition, there is very limited information on the changes in nutritional risk over time [57,63].

## 5. Conclusions

The assessment of the nutritional risk of stroke patients has received less attention than it deserves if one considers the expected impact of the disease on nutritional status and the fact that most patients are elderly and possibly suffer from comorbidities. In other words, despite its relevance to both prevention and treatment, the implementation of best practices in nutritional care is often not adequately considered in the multidisciplinary approach to the diagnosis and management of patients affected by acute or chronic diseases.

This literature review indicates that different screening tools/procedures have been applied to stroke patients with varying prevalence of nutritional risk and clear relationships with clinical outcomes in the short/long term. Indeed, there have been no consistent attempts to compare the usefulness and reliability of the different tools/procedures.

Research inquiries that could be investigated and considered in future studies include the identification of the most appropriate approaches to assessing nutritional risk among stroke patients in the acute and the post-acute phase of disease or during rehabilitation; the evaluation of nutritional risk during a stay in a hospital or rehabilitation unit in relation to different nutritional treatments; and the use of nutritional screening in well-defined nutrition care protocols.

## Figures and Tables

**Table 1:** Papers evaluating nutritional risk among stroke patients with the corresponding screening tools used in each study (continued on next page).

First Author	Journal	Year	Country	Setting	Type of Stroke	MUST	NRS -2002	MNA-SF	GNRI	PNI	CONUT
Akimoto [14]	*Ann. Nutr. Metab.*	2021	Japan	Hosp	I				●		●
Bao [15]	*Neuropsychiatr. Dis. Treat.*	2022	China	Hosp	I				●	●	●
Bise [16]	*J. Nutr. Health Aging*	2022	Japan	Re	I/H *				●		
Cai [17]	*Eur. J. Clin. Nutr.*	2020	China	Hosp	I		●				●
Chang [18]	*Sci. Rep.*	2022	Taiwan	Re	I/H			●			
Chen [19]	*Front. Nutr.*	2022	China	Hosp	I		●				
Chen [7]	*Nutrients*	2022	China	Hosp	I/H				●		
Eto [20]	*J. Stroke Cerebrovasc. Dis.*	2022	Japan	Hosp	I						●
Gomes [21]	*J. Stroke Cerebrovasc. Dis.*	2015	UK	Hosp	I/H	●					
Gon [22]	*J. Stroke Cerebrovasc. Dis.*	2020	Japan	Hosp	I						●
Gon [23]	*J. Clin. Neurosci.*	2022	Japan	Hosp	H						●
Fluck [24]	*Nutr. Clin. Pract.*	2021	UK	Hosp	I/H	●					
Han [25]	*Nutrients*	2022	China	Hosp	I					●	●
Hao [26]	*J. Clin. Lab. Anal.*	2022	China	Hosp	I						●
Hua [27]	*Nutrients*	2022	China	Hosp	I				●		
Huang [28]	*J. Clin. Neurosci.*	2022	China	Hosp	I					●	
Irisawa [29]	*Nutrients*	2020	Japan	Re	NR				●		
Kamimoto [30]	*J. Stroke Cerebrovasc. Dis.*	2022	Japan	Re	I/H *				●		●
Kang [31]	*PLoS ONE*	2020	Korea	Hosp	I				●		
Kokura [32]	*J. Stroke Cerebrovasc. Dis.*	2016	Japan	Hosp	I/H *				●		
Kokura [33]	*Nutrition*	2020	Japan	Hosp	I/H						●
Kokura [34]	*JMA J.*	2022	Japan	Hosp	I/H			●	●		●
Lee [35]	*NeuroRehabilitation*	2021	Korea	Hosp	I			●			
Lee [36]	*Nutrients*	2021	Korea	Hosp	I				●		
Lee [37]	*Int. J. Rehabil. Res.*	2022	Korea	Re	I/H						●
Liu [38]	*Comp. Math. Meth. Med.*	2022	USA	Hosp	NR					●	
Liu [39]	*Althern. Ther.*	2022	China	Hosp	I		●				
Lopez Espuela [40]	*Biol. Res. Nurs.*	2019	Spain	Hosp	I/H						●
Luo [41]	*NMCD*	2022	China	Hosp	I					●	●
Maruyama [42]	*J. Stroke Cerebrovasc. Dis.*	2018	Japan	Re	I/H *				●		
Matsumoto [43]	*Nutrients*	2022	Japan	Re	I/H *				●		
Naito [44]	*Nutrition*	2018	Japan	Hosp	I					●	●
Naito [45]	*J. Neurol. Sci.*	2020	Japan	Hosp	I				●		●
Nezu [46]	*J. Atheroscler. Thromb.*	2022	Japan	Hosp	I				●		●
Nii [47]	*J. Stroke Cerebrovasc. Dis.*	2016	Japan	Re	I/H *				●		
Nishioka [48]	*Clin. Nutr.*	2017	Japan	Re	I/H *				●		
Nishioka [49]	*Eur. J. Clin. Nutr.*	2017	Japan	Re	I/H *			●			
Nishioka [50]	*J. Hum. Nutr. Diet*	2020	Japan	Re	I/H *	●		●	●		
Nishioka [51]	*J. Hum. Nutr. Diet*	2021	Japan	Re	I/H *			●			
Nishiyama [52]	*Neurol. Med. Chir.*	2019	Japan	Re	I/H *			●			
Nozoe [53]	*J. Stroke Cerebrovasc. Dis.*	2016	Japan	Hosp	I/H				●		
Nozoe [54]	*Clin. Neurol. Neurosurg.*	2021	Japan	Hosp	I/H				●		
Nozoe [55]	*Nutrients*	2021	Japan	Hosp	I/H				●		
Qi [56]	*World Neurosurg.*	2019	China	Hosp	SAH					●	●
Sato [57]	*J. Stroke Cerebrovasc. Dis.*	2019	Japan	Hosp	I/H				●		
Sato [58]	*J. Stroke Cerebrovasc. Dis.*	2022	Japan	Hosp	I/H				●		
Scrutinio [59]	*Arch. Phys. Med. Rehabil.*	2020	Italy	Re	I					●	
Shiga [60]	*Nutrition*	2020	Japan	Hosp	I						●
Shiga [61]	*Intern. Emerg. Med.*	2022	Japan	Hosp	H						●
Shimizu [62]	*J. Am. Med. Dir. Assoc.*	2019	Japan	Re	I/H *			●			
Siotto [63]	*Nutrients*	2022	Italy	Re	I/H			●	●		
Song [64]	*Front. Nutr.*	2022	China	Hosp	I				●		
Sremanakova [65]	*J. Stroke Cerebrovasc. Dis.*	2019	UK	Hosp	I/H	●					
Tang [66]	*Front. Aging Neurosci.*	2022	China	Hosp	I (tr)				●	●	●
Xiang [67]	*Front. Neurol.*	2020	China	Hosp	I (tr)					●	●
Yuan [68]	*Clin. Nutr.*	2021	China	Hosp	I				●	●	●
Yuan [69]	*Front. Nutr.*	2022	China	Hosp	I						●
Zhang [70]	*Clin. Nutr.*	2021	China	Hosp	I	●	●		●		●
Zhang [71]	*Stroke*	2022	China	Hosp	I				●	●	●
Zhu [72]	*Br. J. Nutr.*	2021	China	Hosp	H						●

Screening tools: MUST = Malnutrition Universal Screening Tool; NRS-2002 = Nutritional Risk Screening 2002; MNA-SF = Mini Nutritional Assessment-short form; GNRI = Geriatric Nutritional Risk Index; PNI = Prognostic Nutritional Index; CONUT = Controlling Nutritional Status. Setting: Hosp = hospital (ward, stroke unit, or hyperacute stroke unit); Re = rehabilitation (hospital rehabilitation ward/unit or rehabilitation hospitals/centers); tr = patients treated with Intravenous Thrombolysis. Type of stroke: I = ischemic; H = hemorrhagic (* = specifically indicating the presence of SAH patients); SAH = subarachnoid hemorrhage; NR = data not reported. Journal: NMCD = Nutrition, Metabolism and Cardiovascular Diseases. ● = nutritional screening tool used.

**Table 2:** Principal outcomes related to nutritional risk (various screening tools) among stroke patients.

	Mortality	Disability/ Independence in ADL	Infections	LOS	Dysphagia	Cognitive Impairment
**MUST**	**HOSP** Fluck [24] Gomes [21] Sremanakova [65] Zhang [70]	**HOSP** Fluck [24] Zhang [70] **RE** Nishioka [50]	**HOSP** Fluck [24] Sremanakova [65]	**HOSP** Gomes [21] Sremanakova [65]		
**NRS-2002**	**HOSP** Cai [17] Zhang [70]	**HOSP** Cai [17] Zhang [70]	**HOSP** Cai [17] Chen [19]	**HOSP** Cai [17]	**HOSP** Liu [38]	
**MNA-SF**		**HOSP** Kokura [34] Lee [35] **RE** Chang [18] Nishioka [49] Nishioka [50] Nishioka [51] Nishiyama [52]			**RE** Chang [18] Shimizu [62]	**RE** Chang [18] Nishioka [51]
**GNRI**	**HOSP** Chen [7] Tang [66] Yuan [68] Zhang [68] Zhang [69] **RE** Maruyama [42]	**HOSP** Akimoto [14] Kang [31] Kokura [32] Kokura [34] Luo [41] Naito [45] Nezu [46] Nozoe [54] Nozoe [55] Sato [57] Zhang [70] Zhang [71] **RE** Bise [16] Irisawa [29] Kamimoto [30] Nii [47] Nishioka [48] Nishioka [50]	**HOSP** Akimoto [14] Song [64] **RE** Maruyama [42] Nishioka [48]	**HOSP** Akimoto [14]	**RE** Bise [16] Matsumoto [43] Nishioka [48]	**HOSP** Lee [37]
**PNI**	**HOSP** Liu [38] Tang [66] Yuan [68] Zhang [71]	**HOSP** Luo [41] Qi [56] Xiang [67] **RE** Scrutinio [59]				
**CONUT**	**HOSP** Cai [17] Hao [26] Lopez Espuela [40] Tang [66] Yuan [68] Zhang [70] Zhang [71] Zhu [72]	**HOSP** Akimoto [14] Cai [17] Eto [20] Gon [22] Gon [23] Kokura [33] Kokura [34] Luo [41] Naito [44] Naito [45] Nezu [46] Qi [56] Shiga [60] Shiga [61] Xiang [67] Zhang [70] Zhang [71] Zhu [72] **RE** Kamimoto [30]	**HOSP** Akimoto [14] Cai [17] Hao [26] Zhu [72]	**HOSP** Akimoto [14] Kokura [33] Shiga [60] Yuan [68] Zhang [70] Zhu [72]		

MUST = Malnutrition Universal Screening Tool; NRS-2002 = Nutritional Risk Screening 2002; MNA-SF = Mini Nutritional Assessment-short form; GNRI = Geriatric Nutritional Risk Index; PNI = Prognostic Nutritional Index; CONUT = Controlling Nutritional Status; ADL = activity of daily living; LOS = length of hospital stay. Setting: HOSP = hospital (ward, stroke unit, or hyperacute stroke unit); RE = rehabilitation (hospital rehabilitation ward/unit or rehabilitation hospitals/centers).

## Data Availability

Not applicable.
